# Computerized multi-domain cognitive training reduces brain atrophy in patients with amnestic mild cognitive impairment

**DOI:** 10.1038/s41398-019-0385-x

**Published:** 2019-01-31

**Authors:** Haifeng Zhang, Zhijiang Wang, Jing Wang, Xiaozhen Lyu, Xiao Wang, Ying Liu, Xiangzhu Zeng, Huishu Yuan, Huali Wang, Xin Yu

**Affiliations:** 1 Peking University Institute of Mental Health (Sixth Hospital), Beijing, 100191 China; 2 National Clinical Research Center for Mental Disorders & Key Laboratory of Mental Health, Ministry of Health, Peking University, Beijing, 100191 China; 3 Beijing Municipal Key Laboratory for Translational Research on Diagnosis and Treatment of Dementia, Beijing, 100191 China; 4 Peking University Third Hospital, Beijing, 100191 China

## Abstract

The present study aimed to explore the effect of computerized multi-domain cognitive training (MDCT) on brain gray matter volume and neuropsychological performance in patients with amnestic mild cognitive impairment (amnestic MCI). Twenty-one patients with amnestic MCI participated in a computerized MDCT program. The program targeted a broad set of cognitive domains via programs focused on reasoning, memory, visuospatial, language, calculation, and attention. Seventeen Participants completed the intervention and all completed a battery of neuropsychological tests to evaluate cognitive function while 12 out of 17 underwent 3 T MRI scanning before and after the intervention to measure gray matter (GM) volume. We examined correlations between the changes in neuropsychological scores and GM volumes across participants after the intervention. After training, we observed significant increases in GM volume in the right angular gyrus (AG) and other parietal subareas near the intraparietal sulcus (*p* < 0.05, FWE-corrected, 10000 permutations). However, we found no significant changes in neuropsychological test scores (*p* > 0.05). A correlation analysis revealed positive correlations between the changes in GM volume in the right AG and scores in the immediate recall component of the Hopkins Verbal Learning Test-Revised (HVLT-R) (*r* = 0.64, *p* *=* 0.024) and the Brief Visuospatial Memory Test–Revised (BVMT-R) (*r* = 0.67, *p* *=* 0.016). Our findings indicate that a computerized MDCT program may protect patients with amnestic MCI against brain GM volume loss and has potential in preserving general cognition. Thus, our non-pharmacological intervention may slow the rate of disease progression.

## Introduction

Dementia is a progressive neurocognitive disorder characterized by deteriorating cognition, behavior, and the ability to perform everyday activities, and currently represents a major public health challenge^1^. Globally, dementia is currently estimated to affect 5–8% of people aged 60 years and above^1^. However, there is no cure for dementia, and to date, no treatments have been able to recover function in affected individuals. Recently, researchers have begun to focus on delaying the progress or reducing the incidence of dementia at its preclinical stages^2^. Mild cognitive impairment (MCI) is a critically preclinical state and a proximal risk factor for dementia. Epidemiological studies have shown that 8–15% of individuals with MCI will progress to dementia every year^3^, and up to 80% will progress to dementia within six years^4^. A group of MCI patients with memory impairment, namely amnestic MCI, is the most common subtype. Amnestic MCI is the typical prodromal stage of dementia and carries a high conversion risk to Alzheimer’s disease (AD)^4,5^. Thus, it is of vital importance to intervene at the stage of amnestic MCI.

Recently, computerized cognitive training has received considerable attention as a non-pharmacological MCI intervention because of its safety, affordability, and scalability in maintaining cognition in older adults^6^. Cognitive training usually refers to a series of repeated and standardized tasks with inherent challenges that target specific cognitive domains^7^. It is based on the theory of neuroplasticity, which holds that the brain adaptively modifies its structure and function in response to external or internal stimulation throughout life^8^. Indeed, neuroplasticity occurs in late life and throughout age-related neurodegenerative disorders, as well as during childhood^9–11^. Observational studies have suggested that positive participation in cognitive training activities is associated with greater gray matter volume and higher cognitive function in both older adults and populations at high risk of developing AD^12–14^. These findings may inspire researchers to design effective intervention protocols for improving cognition in such populations.

Accumulating evidence indicates that cognitive training could benefit cognition in patients with MCI and early AD^6,15–17^. Most previous studies conducted single-domain cognitive training in patients with MCI, such as that focused on episodic memory^15,16,18–20^, working memory^17,21,22^, and speed of processing^23^. For example, Savulich et al. reported that after 4 weeks of novel memory-game training on an iPad, patients with amnestic MCI exhibited a robust improvement in episodic memory function^16^. Belleville et al. reported that eight weeks of weekly episodic memory training significantly enhanced subjective memory and well-being in patients with MCI^8,15^. However, although memory loss is the main symptom in patients with amnestic MCI, other cognitive domains such as verbal fluency, language, attention, and executive function are often affected, and these have been associated with the observed memory deficits^24^.

Thus, compared with single-domain training, multi-domain cognitive training (MDCT) included both memory and non-memory domains (e.g., execution, attention, etc.), and would have broader beneficial effects on cognition and brain measurements in patients with amnestic MCI. These effects have been found in normal aging populations, probably due to the cooperation between different domains in favor of protecting or improving overall cognition^13,25,26^. For example, Li and colleagues performed an MDCT, which combined memory strategy and executive function training and found a larger training effect in memory and broader training effects in non-memory outcomes than pure memory training^26^. On changes in brain measurements, Cao and colleagues administered a 3-month MDCT program targeting memory, reasoning, and problem-solving in healthy older adults. They found that the brain functional connectivity between three higher cognitive functional networks, i.e., the default mode network (DMN), the salience network, and the central executive network (CEN), was enhanced after one year^27^.

However, to the best of our knowledge, no MDCT interventions have examined changes in brain gray matter (GM) volume in patients with amnestic MCI. In this study, we designed an MDCT intervention protocol that engaged a broad range of cognitive domains via tasks involving reasoning, memory, visuospatial skill, language, calculation, and attention, delivered to patients with amnestic MCI via a tablet computer. The aim is to examine the effect of the computerized MDCT program on brain GM volume and cognitive performance in the participants.

## Material and methods

### Study design

The present study used a self-control design to investigate the effect of the MDCT program on brain GM volume and neuropsychological performance in patients with amnestic MCI. The intervention consisted of a 12-week training program targeting a wide range of cognitive domains via programs requiring reasoning, memory, visuospatial skill, language, calculation, and attention. The programs were delivered twice per week. We conducted a neuropsychological assessment and MRI scan before and after the training program. Evaluators were blind to the intervention type.

### Participants

Twenty-seven patients with amnestic MCI were recruited from Dementia Care & Research Center of Peking University Institute of Mental Health (DCRC-PKUIMH) between May 2015 and September 2015. Twenty-one patients met the inclusion criteria and signed an informed consent form. They then completed a standardized neuropsychological assessment at DCRC-PKUIMH and were scheduled to undergo MRI scanning at Peking University Third Hospital. Four patients withdrew from the MDCT program because of personal reasons. MRI scanning for 12 participants was conducted using a 3.0T General Electric (GE) Magnetic Resonance Imaging (MRI) 750 (Boston, Massachusetts, USA.), and that for four patients was done using a 3.0T Siemens Magnetom Trio (Munich, Germany). One patient could not stay in the MRI scanner because she had metal body implants. While 17 patients completed the training program, we used the neuropsychological assessment data from all the 17 patients and MRI data from the 12 patients who completed the intervention and were scanned in the 3.0 T GE MR750.

All participants met Petersen’s amnestic MCI criteria, specifically: (a) memory problems, confirmed by an informant, (b) preserved general cognitive function (mini-mental state examination (MMSE)^28^ score of >24/30), (c) intact activities of daily living (an ADL score of ≤26), and (d) do not meet the diagnosis of dementia (according to the 10th International Statistical Classification of Diseases and Related Health Problems (ICD-10)^29^ and National Institute of Neurological and Communicative Disorders and Stroke and Alzheimer’s Diseases and Related Disorders Associations (NINCDS-ADRDA)^30^). Other inclusion criteria were as follows: (1) age ≥ 55 years, (2) right-handed, (3) primary school education (≥5 years), (4) a Clinical Dementia Rating (CDR) = 0.5, and (5) a Hamilton Depression Scale (HAMD) score of <12.

Exclusion criteria were Axis I psychiatric disorders listed in the Diagnostic and Statistical Manual of Mental Disorders 4th edition (DSM-IV)^31^, neurological pathology, alcohol or substance abuse/dependence, head injury with loss of consciousness that could affect cognitive function and/or psychiatric behavior, current pharmaceutical regiment, including cognitive enhancers or antidepressants, and any physical condition that could preclude regular attendance and full participation in the intervention program. The present study was approved by the ethics committee of Peking University Institute of Mental Health (Sixth Hospital), Beijing, China. All participants were fully informed regarding the study protocol and provided written informed consent.

### Interventions

As mentioned, the MDCT intervention covered a broad range of cognitive domains with tasks that involved reasoning, memory, visuospatial skills, language, calculation, and attention. The cognitive training program was co-developed with Beijing Neowave Technology Co., Ltd, and run on a tablet computer. The intervention was carried out at the senior center of Peking University Health Science Center and led by trainers who were able to solve technical issues associated with the operation of the tablet computer. The trainers were not permitted to engage in any discussion with the participants about how to finish the tasks. The whole intervention comprised 24 training sessions delivered twice per week throughout 12 weeks. Each session included tasks that focused on three cognitive domains, and the participants spent 20 min engaged in each task in a session. There were two different 1-hour sessions per week for a total of 2 h with six different cognitive domains. Each participant experienced the same training schedule from the very first session to the last session.

Each task contained 3–5 different levels of difficulty, with six trials in each difficulty level. If a participant answered correctly in four or more trials, the difficulty level would be increased; conversely, if a participant answered incorrectly in more than two trials, the difficulty level in the next trial would be decreased. We modulated the task difficulty to maintain the degree of challenge and maximize participant performance. During the study, all participants did not receive cognitive enhancers that are approved by the China Food and Drug Administration (CFDA) for treatment of Alzheimer’s disease.

### Cognitive assessment

We assessed cognition both at the baseline and after the 12-week MDCT intervention. Based on a survey of neuropsychological assessment^32^, we designed a comprehensive cognitive test battery that examined memory, reasoning, visuospatial ability, language, attention, processing speed, and executive functioning. The individual tests are listed below. Global cognition was assessed via the MMSE^28^ and Montreal Cognitive Assessment (MoCA)^33^. The entire cognitive assessment took about 120 min.

Memory was evaluated via the Hopkins Verbal Learning Test-Revised (HVLT-R)^34,35^. The speed of processing was examined using the Trail Making Test A (TMT-A)^36^. Visuospatial ability was examined using the Brief Visuospatial Memory Test-Revised (BVMT-R)^37^. Language function was examined using the verbal fluency test for animal naming^38,39^. Attention and working memory were examined using the Digit span test and the Space span test^38^. Executive function was assessed via a 100-stimulus version of the Stroop color and word test. Reasoning ability was assessed via a picture completion test.

### MRI acquisition and preprocessing

We conducted MRI scans in 12 patients using a 3.0T GE MRI 750 with an eight-channel sensitivity encoding head coil (SENSE factor = 2.4) and parallel imaging at the Peking University Third Hospital Neuroimaging Centre. Structural T1-weighted MRI images (Flip angle = 15°, field of view (FOV) = 240 × 240 mm; slice thickness = 1 mm, repetition time (TR) = 4.8 ms, echo time (TE) = 2.04 ms, inversion tome = 400 ms, acquisition matrix = 256 × 256) were obtained at the baseline and after the 12-week intervention procedure.

To measure brain GM volume, we performed voxel-based morphometry (VBM) analysis for structural images using the DARTEL algorithm^40^ in Statistical Parametric Mapping (SPM12, http://www.fil.ion.ucl.ac.uk/spm). Spatially normalized GM maps were modulated via the Jacobian determinant of the deformation field and then corrected for individual brain sizes. The modulated GM maps were then resampled to 1.5-mm isotropic voxels and smoothed using an 8-mm full-width at half maximum (FWHM) Gaussian kernel.

### Statistical analysis

We analyzed the efficacy of the MDCT using the full set of amnestic MCI patients (17 in total). The multiple-domain and global cognition scores were assessed using paired *t* tests. We used Statistical Package for Social Sciences (SPSS) software (version 20.0 for Windows, SPSS Inc., Chicago, IL) for all analyses. All the cognitive variables were normally distributed and were assessed using the paired *t* test. The Shapiro–Wilk test was used for the test of normality of differences of pairs of cognitive variables. The null-hypothesis of this test is that the data are normally distributed. If the *p* value is less than the alpha value, then the null hypothesis is rejected, and there is evidence that the data tested are not normally distributed. And the alpha value was set at 0.05 in the present study.

We investigated differences in the brain GM volume before vs. after the MDCT program over the 12 patients for whom we obtained MRI data at the 3.0T GE MRI 750. We used Statistical nonParametric Mapping software (SnPM13, http://warwick.ac.uk/snpm), which provides an extensible framework for non-parametric permutation tests based on the general linear model as well as pseudo-t-statistics for independent observations. We performed a pseudo-voxel-level paired *t* test to compare the GM volume maps before (baseline) vs. after the MDCT program within a group-averaged GM mask (i.e., GM probability > 0.2). The result was then thresholded at a cluster-level corrected threshold of *p* < 0.05 (*n* = 10000 permutations, FWE-corrected) with a cluster-forming threshold at the voxel level *p* < 0.001.

We performed correlation analyses for regional changes in GM volume and changes in neuropsychological test scores to assess whether brain structural changes were associated with changes in cognitive performance before vs. after the MDCT intervention.

## Results

**Fig. 1:**
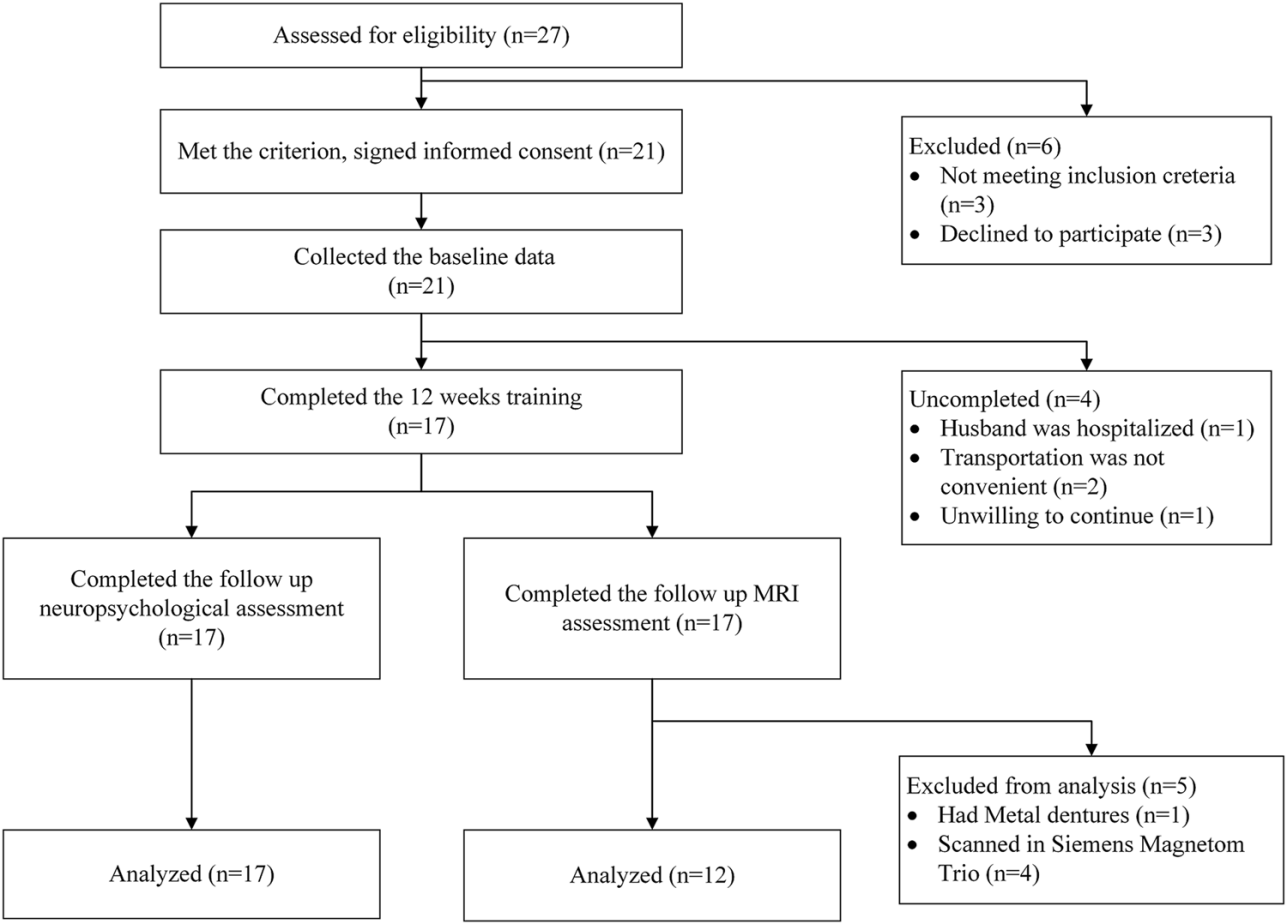
The flow chart showing the study procedure

From May to September 2015, 17 (81%) patients with amnestic MCI completed the 12-week MDCT program with the associated global cognitive test and neuropsychological test battery. Of them, 12 patients were scanned using a 3.0 T GE MR750; four patients were scanned using a 3.0T Siemens Magnetom Trio, and the last patient could not be scanned because she had body metal implants (see Fig. 1). The average participant compliance was 88% (33–100%) for the total 24 training sessions.

The characteristics of all the participants at baseline are shown in Table 1. Most of the participants were female, were about 75 years old, and had a relatively high education level (college level). General cognition was preserved, activities of daily living were intact, and the participants were not depressed (See Table 1).

**Table 1:** Baseline Characteristics of research participants who completed neuropsychological tests and imaging data collection

Variables	Completing neuropsychological test (*n* = 17)	VBM (*n* = 12)
Gender (male/female)	6/11	4/8
Age (years)	75.2 ± 3.8	74.8 ± 3.6
Education (years)	13.6 ± 3.3	13.9 ± 2.7
MMSE	27.4 ± 2.2	26.8 ± 2.2
ADL	20.9 ± 2.3	21.3 ± 2.8
HAMD	2.4 ± 3.7	2.4 ± 4.4

*MMSE* mini-mental state examination, *HAMD* Hamilton depression scale, *ADL* activities of daily life, *VBM* voxel-based morphometry

### Changes in cognitive function

Table 2 shows the neuropsychological test scores before (baseline) and after the MDCT intervention, as well as *p* values that show the significance levels of the reported differences. We did not observe any significant differences in neuropsychological test scores for the global cognitive test or specific cognitive domains after the 12-week MDCT intervention (*p* > 0.05).

**Table 2:** Changes in cognitive performance after a 12-week MDCT intervention

	Baseline	3 months	*P* value
*Overall cognition*			
MMSE	27.35 ± 2.23	27.06 ± 2.97	0.589
MoCA	22.47 ± 4.47	21.88 ± 4.29	0.347
*Memory*			
HVLT-R immediate recall Trial 1	5.53 ± 1.74	4.94 ± 1.85	0.106
HVLT-R immediate recall Trial 2	7.18 ± 1.78	6.82 ± 1.55	0.463
HVLT-R immediate recall Trial 3	7.88 ± 1.80	8.00 ± 1.23	0.805
HVLT-R immediate recall total	20.59 ± 4.77	19.76 ± 3.62	0.456
HVLT-R delayed recall	5.12 ± 2.90	5.35 ± 2.93	0.753
HVLT-R retention (%)	59.94 ± 33.82	63.91 ± 34.06	0.656
HVLT-R recognition discrimination Index	8.12 ± 3.04	7.76 ± 3.58	0.231
*Speed of processing*			
Trail Making Test Part A (s)	79.06 ± 48.57	76.18 ± 39.76	0.600
*Visuospatial ability*			
BVMT-R immediate recall total score	18.35 ± 7.83	16.53 ± 8.74	0.286
BVMT-R delayed recall score	6.88 ± 3.50	6.88 ± 3.84	1.000
*Language*			
Verbal Category Fluency	16.59 ± 4.05	16.65 ± 4.51	0.956
*Attention and working memory*			
Digit Span total score	15.47 ± 4.35	16.24 ± 3.44	0.389
Space Span total score	13.41 ± 2.83	13.41 ± 3.94	1.000
*Executive function*			
Stroop-word	65.94 ± 10.18	65.88 ± 10.02	0.968
Stroop-colour	51.75 ± 9.42	53.06 ± 7.59	0.402
Stroop- Color-Word	24.88 ± 7.44	24.75 ± 8.13	0.943
Golden’s Interference Score	−3.94 ± 7.08	−4.44 ± 7.67	0.764
*Reasoning*			
Modified Picture Completion	7.41 ± 1.66	6.88 ± 1.76	0.058

*MMSE* mini-mental state examination, *MoCA* montreal cognitive assessment, *HVLT-R* Hopkins verbal learning test–revised, *BVMT-R* Brief Visuospatial Memory Test–revised

### Changes in GM volume

**Fig. 2:**
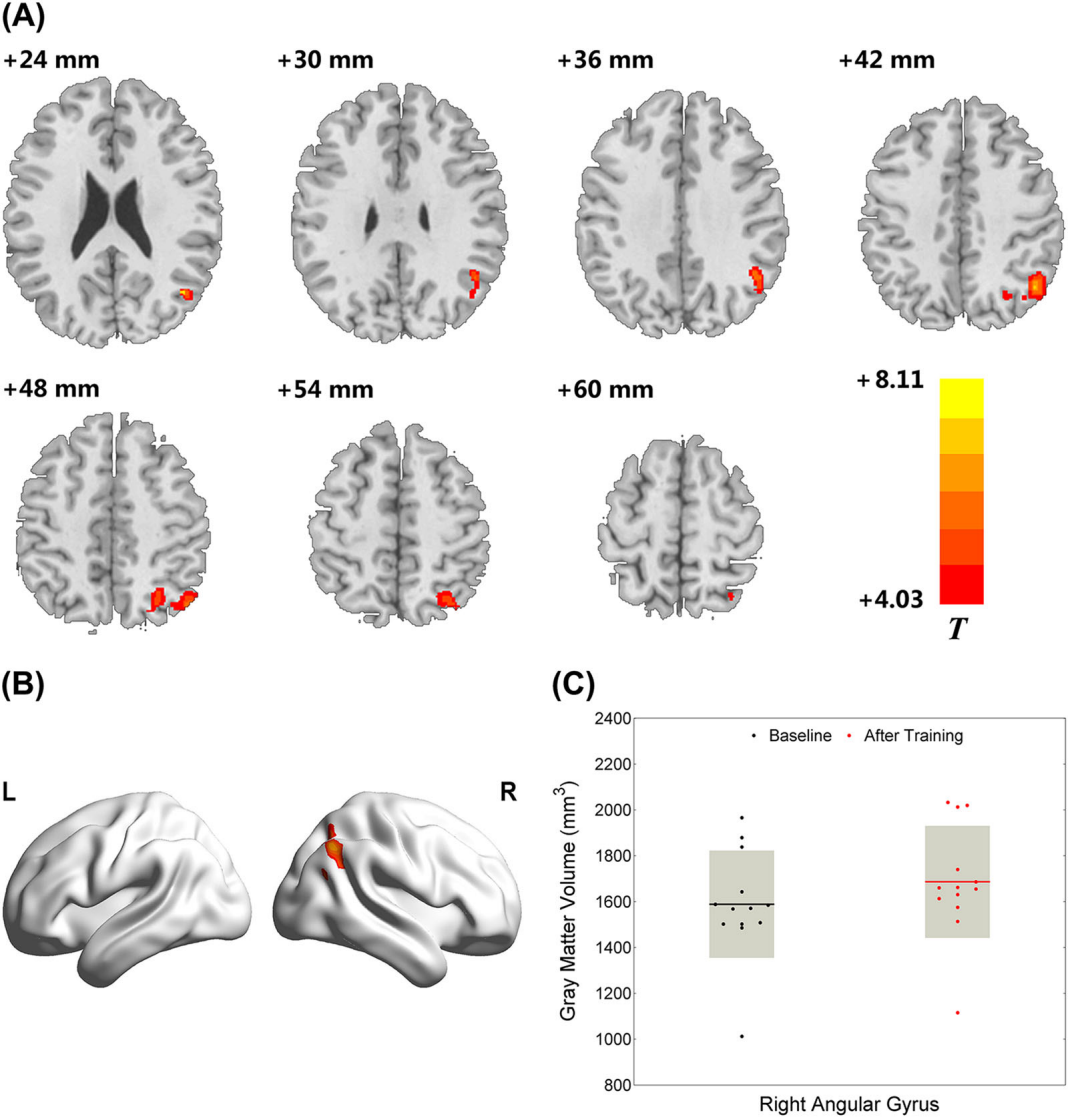
Right angular gyrus (AG) GM volume increased after the multi-domain cognitive training (MDCT) program (*p* < 0.05, FWE-corrected, 10000 permutation). **a** An axial view of the brain which shows the distribution of significantly increased gray matter volume after the intervention; **b** A 3D brain view which shows the region with significantly increased gray matter volume after intervention; **c** A bar showing gray matter volume of right angular gyrus at the baseline and after the intervention

After training, we observed significant increases in GM volume in the right angular gyrus (AG), which partly extended to other parietal subareas near the intraparietal sulcus (see Fig. 2; BA 39, cluster size = 1312 voxels, peak MNI coordinate = (43.5, −64.5, 25.5), *p* < 0.05, FWE-corrected, 10000 permutations). In total, we observed an average increase of 6.14% in GM volume in the right AG area across all participants.

**Fig. 3:**
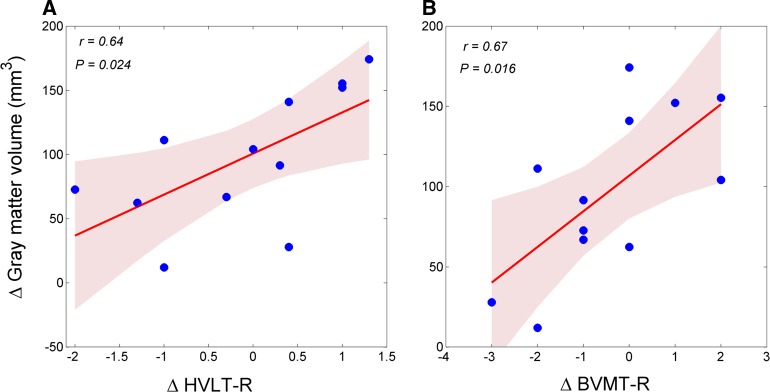
Correlations between changes in gray matter volume and neuropsychological test scores. **a** Positive correlation between right AG volume and score on the immediate total recall component of the Hopkins Verbal Learning Test-Revised (HVLT-R); Shaded areas delineate the confidence interval of 95%. **b** Positive correlation between right AG volume and the immediate recall total component of the Brief Visuospatial Memory Test–Revise (BVMT-R); Shaded areas delineate the confidence interval of 95%

### Correlation analysis between GM volume and cognitive function

We found significantly positive correlations between changes in GM volume in the right AG area and scores on the immediate recall component of the HVLT-R (*r* = 0.64, *p* = 0.024) and BVMT-R (*r* = 0.67, *p* = 0.016) across the 12 patients who participated in the intervention activities (Fig. 3).

## Discussion

In the present study, we designed an MDCT intervention and administered it to individuals with amnestic MCI. The program was delivered on a tablet computer twice a week for 12 weeks. We found a significant increase in GM volume, mainly in the right AG. The increase of the AG volume was positively correlated with an improvement in immediate memory and visuospatial memory abilities. Our main findings indicate that the MDCT has potential in assisting patients with amnestic MCI to preserve general cognition and resist brain structural atrophy.

According to previous studies, MDCT programs could drive a broader effect on maintenance or improvement of overall cognition compared with single-domain (e.g., memory) training programs due to cognitive cooperation across different domains^13,25,26,41^. Our MDCT intervention targeted a wide range of cognitive domains involving reasoning, memory, visuospatial skill, language, calculation, and attention to investigate the efficacy of the intervention on both cognition and neural changes (via brain volume). Here, all task trials included adaptive modulations in task difficulty to maintain a degree of challenge and maximize the training effect. As results, we found that MDCT-related brain GM volume significantly increased, mainly in the right AG area, although this extended to other parietal subareas near the intraparietal sulcus. This finding may support the brain reserve hypothesis^42^ and the hypothesis that neuroplasticity occurs even in individuals with age-related neurodegenerative disorders^8–10^. Previous multi-disciplinary cognitive training studies have reported changes in GM volume or functional connectivity in patients with MCI or older adults after training. However, the protocols of these studies were not limited to cognitive training, but also included physical^43–45^ and lifestyle manipulations^43^ such as physical exercise, meditation, behavioral/music therapy, education regarding a Mediterranean diet, stress reduction strategies, and assistance with improving sleep hygiene. Such training was likely to address a wide set of dementia-related risk factors across the human body, but could be classified more as lifestyle changes rather than as a non-pharmacological intervention as well. A recent study appeared that pure physical activity in healthy old adults over 12 weeks also have protective effects on cognitive health^46^. In the present study, we chose to narrow our focus to better examine the mechanisms underlying post-training brain changes.

The observed increase in GM volume in the right AG might serve as an indicator of the efficacy of the training program, specifically the effect of multi-cognitive-domain functional cooperation. According to previous studies, the AG and surrounding area is widely involved in some cognitive processes related to language, number processing, spatial cognition, memory retrieval, and attention, suggesting that it might serve as a cross-modal integrative hub^47^. Indeed, brain functional network studies have identified the AG as a highly-connected hub node of networks that plays a vital role in integrative information processing and adaptive behaviors^48^. Our MDCT presented multi-domain cognitive stimuli, which may have repeatedly activated the AG in its hub-like role, integrating diverse information associated with different cognitive functions. Findings of a recent study provided further evidence for the AG as a core of the DMN. Specifically, the authors reported that the AG greatly contributed to global information integration in response to increasing environment/task demands^49^.

Moreover, the AG region is normally predominantly disrupted in patients with amnestic MCI and AD, not only regarding network performance^50,51^ but also structural GM volume^52^. Further, patients with amnestic MCI who lastly progressed to AD had more severe GM atrophy than those stable MCI patients^52,53^. Therefore, we propose that intervention-induced changes in the AG region might help to delay the progression of dementia in the patients with amnestic MCI.

Hub regions of brain networks (e.g., AG) exhibit higher cerebral blood flow and metabolic rates in healthy participants, in a manner that is positively correlated with task load^54^. Concerning the relationship between atrophy and metabolic brain alterations in AD, Chetelat and colleagues (2008) reported that hypometabolism exceeded atrophy in the AG and other abnormal brain regions in patients with AD. They suggested that some functional alteration (e.g., metabolic, chemical, or molecular) may occur specifically over and above neuronal/synaptic loss^55^. Thus, special functional activation elicited by cognitive task involvement could induce higher rates of metabolism in the brain, particularly in hub regions that process multi-domain stimuli. Stronger and more diverse activation of neurons in the area activated during MDCT could induce cerebral blood flow and increase metabolism, thus benefiting the AG region regarding functional recovery and protection against structural loss. Indeed, a recent study by Ciarmiello and colleagues (2015) reported a significant increase in brain metabolism in the prefrontal and temporal areas in patients with MCI after a cognitive training intervention (attention, executive function, and working memory)^56^. Therefore, the increase in GM volume in the AG region observed in this study may imply that the MDCT could slow down disease progression in patients with amnestic MCI.

One may argue that the factors other than intervention, such as aging or disease compensation, may potentially contribute to the change of gray matter volume in angular gyrus. However, the existing evidence strongly suggested that these factors result in decrease but not the increase of the gray matter volume. First, though the brain connectivity (white matter) between the different lobes could increase to compensate the deterioration of brain atrophy in aging population^57^, the gray matter volume consistently decline at a rate of about 0.4% in the cognitive stable elderly during follow-up visits^58,59^. Second, previous large sample study has confirmed the gray matter loss of temporoparietal cortex, where the angular gyrus is situated, during the progression of amnestic MCI^60^. Thus, the volume reduction of AG may be sensitive to the progression of MCI, instead of being preserved during the disease progression. Therefore, the increase of the AG volume could largely be explained by the benefit of cognitive training.

However, in the present study, we did not observe significant changes in neuropsychological test scores before vs. after the intervention. This was probably because of insufficient training duration, consistent with the result of Ciarmiello et al. (2015). As Ciarmiello and colleagues discussed^56^, the training effect was presumably related to the total time of training administered to each participant. Our intervention consisted of 24 h with six cognitive domains, and thus both the total and single-domain training duration may not have been sufficient to change clinical performance, which may occur much later than brain structural, morphological, or metabolic changes. Therefore, it may be difficult to find an immediate, specific effect of training on cognitive performance during such a short intervention period. Another possible explanation is that the cognitive outcome measures might not be sensitive to the change of the disease course^60^. Whitwell and Jack Jr et al. also reported that after an 18 months follow-up, the memory, which measured by Auditory-Verbal Learning Test (AVLT), of the progressive MCI group is stable when compared with the baseline data. However, the cognitive function in the participants remained stable over three months and did not decline, which might represent a neuroprotective effect in this population.

Concerning the relationship between changes in the brain and neuropsychological performance, we found positive correlations between the changes in GM volume in the right AG and the neuropsychological scores on the immediate total recall components of the HVLT-R and BVMT-R. These findings suggest that the patients with amnestic MCI benefited from the MDCT intervention and that they have the potential to achieve heightened cognitive performance if the intervention was continued over a longer period. Overall, the intervention may slow down cognitive decline, and prolong the time before the development of dementia. Thus, increased volume of the right AG may be useful as an observable marker for monitoring and testing the efficacy of cognitive training interventions.

To our knowledge, this is the first study to investigate the effect of MDCT, delivered via a tablet computer, on brain GM volume in patients with amnestic MCI. However, there are several limitations to the present study for further considerations. First, our sample size was relatively small, which may lead to underestimation regarding possible improvements in clinical performance. Second, the training duration may have been inadequate, especially for each cognitive domain, with the accumulated time allotted to each domain being only 4 h. It is much less than that provided for other single-domain cognitive training programs^15–17^. It may partly explain why we did not find significant changes in the various cognitive domains. Third, the lack of a control group may be a concern when examining the training effect. Our study was designed as a proof-of-concept study on the brain mechanisms of the cognitive training, to some extent, pre- and post-training comparisons could provide evidence of the potential neural benefits of the intervention. However, it warrants further studies with larger sample size and a control group. Based on the observations in the present study, we have initiated a randomized controlled trial^61^. The ongoing trial will hopefully provide important information on the underlying mechanism of MDCT and its potential role in the prevention of dementia.

## Conclusion

In summary, computerized multi-domain cognitive training may protect against loss of brain GM volume and preserve cognitive functions in patients with amnestic MCI. Thus, this intervention may slow the rate of disease progression. MDCT has potential as a non-pharmacological intervention for dementia.
